# Knee pain and related health in the community study (KPIC): a cohort study protocol

**DOI:** 10.1186/s12891-017-1761-4

**Published:** 2017-09-21

**Authors:** G. S. Fernandes, A. Sarmanova, S. Warner, H. Harvey, K. Akin-Akinyosoye, H. Richardson, N. Frowd, L. Marshall, J. Stocks, M. Hall, A. M. Valdes, D. Walsh, W. Zhang, M. Doherty

**Affiliations:** 1 Academic Rheumatology, Division of Rheumatology, Orthopedics and Dermatology, School of Medicine, University of Nottingham, Nottingham City Hospital, Nottingham, NG5 1PB United Kingdom; 2 Arthritis Research UK Centre for Sports, Exercise and Osteoarthritis, University of Nottingham, Nottingham, NG7 2UH United Kingdom; 3 Arthritis Research UK Pain Centre, University of Nottingham, Nottingham, NG5 1PB United Kingdom

**Keywords:** Knee pain, Osteoarthritis, Phenotypes, Neuropathic pain, Pain Catastrophising, Quantitative sensory testing

## Abstract

**Background:**

The incidence, progression and related risk factors for recent-onset knee pain (KP) remain uncertain. This study aims to examine the natural history of KP including incidence and progression and to identify possible phenotypes and their associated risk factors.

**Methods:**

A prospective community-based cohort of men and women aged 40 years or over within the East Midlands region (UK) will be recruited via a postal questionnaire from their general practices. The questionnaire will enquire about: presence and onset of KP; pain severity (0–10 numerical rating scale (NRS)); pain catastrophizing and neuropathic-like pain (NP) using the painDETECT questionnaires (definite NP scores ≥19–38); risk factors for KP and/or osteoarthritis (OA) (age, body mass index, constitutional knee alignment, nodal OA, index: ring finger length (2D4D) ratio); quality of life (SF12); and mental health (Hospital Anxiety and Depression Scale). Clinical assessments will be undertaken in a sample of 400 participants comprising three groups: early KP (≤3 year’s duration), established KP (>3 years) and no KP. Assessments will include knee radiographs (standing semi-flexed and 30^0^ skyline views); knee ultrasound (synovial effusion, hypertrophy, and Doppler activity); quantitative sensory testing; muscle strength (quadriceps, hip abductor, and hand-grip); balance; gait analysis (GAITrite); and biomarker sampling. A repeat questionnaire will be sent to responders at years 1 and 3. The baseline early KP group will undergo repeat assessments at year 1 (apart from radiographs) and year 3 (with radiographs). Any incident KP individuals identified at year 1 or 3 questionnaires will have clinical and radiographic assessments at the respective time points.

**Discussion:**

Baseline data will be used to examine risk factors for early onset KP and to identify KP phenotypes. Subsequent prospective data, at least to Year 3, will allow examination of the natural history of KP and risk factors for incidence and progression.

**Trial registration:**

The study was registered on the clinicaltrials.gov portal: NCT02098070) on the 14th of March 2014.

## Background

Knee pain (KP) is a very common musculoskeletal condition and is a leading cause of disability in people aged over 50 years [1]. Approximately 1 in 4 people in the UK general population have KP in this age group [2–4], largely attributed to the presence of underlying knee osteoarthritis (OA).

The relationship between KP and knee OA is complex and there is often a marked discordance between structural joint changes and clinical symptoms [5]. Most studies focus on late or established OA in people who may have had pain for many years, and few studies have examined recent onset KP and “early” clinical OA. Although people with more persistent and long-standing troublesome KP are most likely to seek medical attention and subsequently be labelled as having knee OA [6], they often represent the more severe end of the knee pain and knee OA spectrum [7]. However, KP is the malady, not knee OA [8]. There is a need to understand the natural history of knee pain from point of onset through the fluctuations of the pain experience including changes in pain type and severity and eventual progression in some to severe, established daily KP. By understanding the risk factors for early onset KP as part of the spectrum of KP, we have the opportunity to target, treat and manage KP symptoms earlier and more effectively, thus potentially preventing the development of a more painful and functionally impaired joint.

In addition to the deleterious effects of the well-documented structural changes of knee OA [9–11], early KP could acquire characteristics of more severe and even neuropathic-like pain (NP) through central rather than peripheral mechanisms. For example, increased central sensitisation of nociceptive pathways where an enhanced localised pain response spreads from the source to adjacent regions may lead to diffuse and more severe regional KP [12, 13]. Also, there may be ineffective pain inhibitory mechanisms [14] due to impaired conditioned pain modulation (CPM) that in people with KP and/or knee OA could cause not only an enhanced pain response but more diffuse KP and a tendency to other regional body pain. In fact, over a quarter of older adults with chronic symptomatic knee OA present with NP suggesting that neuropathic mechanisms are contributing to the pain experience [15]. Soni et al [16] reported that KP at baseline was predictive of a significantly higher risk of subsequent knee symptoms (9% persistent KP and 29% intermittent KP) and that those with radiographic OA, symptoms of depression and multiple regional pain were more likely to have constant pain while those with inconstant pain reported better function and quadriceps strength. OA is therefore not a static disease with pain as its main symptom evolving over time. It has been suggested that in the early stages KP is more likely to be mild and intermittent, whereas in the later stages pain persistence and intensity increase [17]. However, this relationship is not always linear and one in four people with KP report that pain has been stable or even improved since its onset [18]. Furthermore, it is possible that the effect of risk factors on pain will change as the pain progresses due to changes both in peripheral and central mechanisms of pain production and modulation.

There are several constitutional and biomechanical risk factors associated with knee pain such as age, body mass index, knee injury and knee alignment [19]. However, apart from structural joint changes, other local or more general person-specific factors (for example, pain, genetic polymorphisms or psychosocial factors) appear likely to influence KP experience and severity and to associate with different outcomes [13, 20, 21]. Such factors, however, have been studied rarely in a general population setting. Characteristics include pain type (such as NP or intermittent or constant pain presentations), pain responses (reduced pain pressure thresholds (PPT)), psychosocial factors (sleep patterns, anxiety and depression and pain catastrophising), biomechanical factors (alignment, proprioceptive ability, and muscle strength/weakness), genetic factors and pathophysiological biomarkers measured in blood or urine. Therefore, with the use of a questionnaire and subsequent clinical assessments, we can examine the relationship between different phenotypes and pain severity, functional limitation and quality of life. The ultimate goal is to assist clinicians and health care providers to select the most appropriate intervention for individual patients according to their phenotypic characteristics, to achieve better outcomes with fewer complications from available interventions.

## Objectives:

1. To better understand the natural history of KP
2. To determine the prevalence of different phenotypes of KP (including features suggesting central pain sensitisation) within the general population
3. To determine the relationship between different phenotypes and pain severity, functional limitation and quality of life
4. To identify possible novel associations with each phenotype
5. To examine the change of KP and associated factors over time

## Methods

### Design

This is a prospective general population cohort study. The main design is to obtain questionnaire data from a large proportion of the local adult population aged 40 and over, and to undertake assessments in a sample of respondents at baseline and then at Year 1 and Year 3 follow-up. More prolonged follow-up, as well as additional nested studies, will be considered subject to scientific review, further ethics committee approvals and funding.

### Ethics

All aspects of this study were approved by the Nottingham Research Ethics Committee 1 (NREC Ref: 14/EM/0015) and registered (clinicaltrials.gov portal: NCT02098070).

### Participants

Inclusion criteria: all men and women aged 40 years and over, located on the General Practitioner (GP) register, irrespective of KP status.

Exclusion criteria: inability to give informed consent, terminal or severe mental illness, and pregnant women (for clinical assessments only).

Eligibility will be decided by the health professionals in each general practice using the GP register. The GP register is a log of patients who have registered with their local National Health Service practice, who have lived in the local area (minimum of 24 h) and who have free access to primary care regardless of nationality or immigration status. Regional general practices will be approached via the Clinical Research Network (East Midlands) including Nottinghamshire and Derbyshire. A postal questionnaire will be sent to approximately 40,000 adults. The baseline questionnaire will be accompanied by a covering letter from their general practitioner (GP) introducing the study and the objectives. Participants will be able to read about the study and if willing, complete the questionnaire and return it in an enclosed pre-paid envelope to Academic Rheumatology (University of Nottingham) at Nottingham City Hospital. Return of a completed questionnaire will be taken as implicit consent. At the end of the questionnaire responders will be asked to indicate separately whether or not they would be willing to: (1) receive further information about a single visit to Academic Rheumatology to undergo knee radiographs and other assessments; (2) receive a further similar postal questionnaire in one year’s time; and (3) to receive further information of other future studies related to knee pain and OA.

#### Baseline

### Questionnaire

This will be a community-based questionnaire survey comprising a sample of the general population of Nottingham irrespective of whether they had experienced KP prior to the time of recruitment. The postal questionnaire will be developed based on a review of items in previously published questionnaires [22, 23]. Two pilot questionnaire versions have been evaluated in volunteers, both with and without KP respectively, to identify any problems with content, language or layout as part of patient and public involvement (PPI) groups at the Nottingham University Hospitals NUH Trust.

The questionnaire will be designed to capture detailed information about the individual, their medical history and currently known risk factors for knee pain and knee OA. The questionnaire will include a section on all current medications (prescribed and over the counter) and will include supplements, vitamins and alternative medications. It will also include an open text question on different treatments tried for knee pain (diet, lifestyle, exercise, footwear modifications, etc) as well as which particular treatments have been most helpful for treating knee pain. The painDETECT Questionnaire (PDQ) which has been validated against expert physician-diagnosis of neuropathic pain (NP) in a range of chronic pain conditions will be chosen to identify NP with a focus on KP (PDQ scores of ≥13 as possible NP and ≥19 as definite NP) [24]. Participants will be asked whether they have experienced pain in or around the knee on most days for at least a month ever and whether they have experienced any KP during the past month. Pain experience will be captured using the validated Intermittent and Constant Osteoarthritis Pain (ICOAP) tool [25–27]. Participants will also be asked to rate their current pain severity using a numerical rating scale (NRS) from 0 to 10. A validated screening question will be used to determine the presence of current KP, specifically: “Have you had knee pain for most days of the past one month?” [28–30]. Current KP, as well as pain elsewhere, will be captured using a body pain mannequin [31]. The Pain Catastrophising Scale (PCS) will be used to quantify pain behaviour and particularly determine whether participants had an exaggerated negative orientation towards a noxious stimulus [32]. Individual comorbidities will be self-reported according to a brief checklist, with data dichotomised into individuals suffering a specified disease (e.g. diabetes or fibromyalgia) and those who did not. An open text question will also be included to capture any information on any other diagnosed medical conditions not on the checklist. The use of analgesic medication will also be recorded in the form of any current medication, both prescribed and those over the counter including vitamins, supplements and alternative medicines as well as the duration of consumption. Constitutional knee alignment (in early 20’s), current knee alignment and 2D4D finger ratio (3 patterns) will be self-reported and assessed using validated line-drawings [19, 33]. In using this instrument, participants separately self-report their current and early adult life (early 20’s – presumed to be constitutional) knee alignment as severe varus, mild varus, straight legs, mild valgus or severe valgus. Those with severe or mild varus are categorised as having a varus alignment, those with severe or mild valgus are classed as having a valgus knee alignment and those with straight legs as neutral alignment. Nodal OA will be determined using a validated line diagram [33] and classified as present in those reporting nodes on at least two rays of both hands [34]. A significant knee injury will be defined as “*one which caused pain for most days for at least a three-month period”.* Occupations will be classed as ‘high risk’ for OA based on published evidence [35, 36]. Each listed occupation per participant will be analysed and the data dichotomised into high- or low-risk groups. For participants who state they are unemployed or retired, any previous occupations will be dichotomised into high or low risk based on job type (see Additional file 1) and duration (full time or part time and number of years per position). If no occupations are listed, then these participants will be categorised as ‘low risk’ in terms of OA. Quality of life will be assessed using the SF-12 which will produce a physical component score and mental component score. Anxiety and depression symptoms will be measured using the Hospital and Anxiety Depression Scale (HADS) which has been extensively validated. The optimal cut-off score for the presence of both symptoms is >8 as this has a corresponding sensitivity and specificity of 0.8 [37, 38]. The questions and their timing are summarised in Table 1.

**Table 1:** Measurement of domains and data collection time points for questionnaire data. (* Clinical assessments will be conducted in a sub-sample of participants at baseline with follow-up assessments at Year 3 for all participants and incident KP cases recruited at Year 1 and Year 3)

Section Domains	Questions & Instruments Included	Baseline	1 year	3 years
Demographic & Occupation Data	Date of birth, Gender, Height & Weight & list of main occupations (duration and whether it was part time or full time)			
Medical & Medication History	Diagnosis of any comorbidities such as diabetes, stroke, fibromyalgia. Trauma or significant injury to the lower limbs. All current medication including supplements and alternative medications.			
Knee Pain	Knee Pain presence, diagnosis of knee OA, any surgical interventions, any treatments for knee pain, ICOAP (Intermittent and Constant OA Pain) Pain Detect Questionnaire			
Knee Alignment	Current and Constitutional alignment using line drawings			
Hands	2D4D ratio; OA nodes; family history of OA nodes and knee or hip joint replacement			
Body Pain	Body Pain Mannequin for current body pain Quality of Pain (severity)			
Psychosocial Factors	Hospital Anxiety & Depression Score			
	Sleep scale from Medical Outcomes Survey (from Year 1) Conscientiousness Test (from Year 1) Fibromyalgia Mannequin (from Year 1) Life Orientation Test (LOT) (from Year 1)			
Quality of Life	SF – 12 Illness Attitude Scale Pain Catastrophising Scale			
Clinical Assessments*				
Blood & Urine Sample Collection	Fasting Biomarker Sample collection			
Ultrasound				
Quantitative Sensory Testing				
Radiographs	Bilateral radiographs consisting of PA view of the tibiofemoral compartment and skyline view of the patellofemoral compartment using a Rosen template (standardised views).			
Gait Assessment	Gaitrite and RS Scan for walking speed, cadence, step length, step width and static and dynamic balance			
Muscle Strength	JAMAR hand dynomometer & Nicholas Muscle Testers for hand grip strength and quadriceps/ hip abductor strength respectively.			

### Clinical assessment

From the questionnaire responders, a sample of participants who indicated a willingness to consider undergoing knee radiographs and other assessments will be identified. Three distinct groups of participants (early KP, established KP and no KP) will then be identified based on their questionnaire responses on KP duration and severity. They will be sent a letter of invitation together with a participant information sheet. Those who reply registering their interest and who give contact details will undergo an additional telephone screening prior to being booked into a single hospital appointment. The inclusion criteria for clinical assessment are:

i. Participants with recent-onset KP (*n* = 200) are defined as mild/moderate and/or intermittent KP occurring for the first time in the past 3 years for most days of at least one month, unrelated to obvious major trauma.

Once, the early KP group is recruited, participants from the established KP (ii) and no KP group (iii) will be age and gender matched to this early KP group (i) using the following inclusion criteria:

ii. Participants with established persistent KP (*n* = 100): defined as KP for over 3 years which has been moderate (NRS >6) and/or persistent for most days of the past 3 months, unrelated to obvious major trauma.

iii. Participants with no KP: defined as no KP (n = 100) within the past 5 years.

Respondents to the questionnaire who report total knee joint replacement surgery or major prior knee injury will be excluded - only those with spontaneous (“primary”) KP will be selected for groups (i) and (ii).

Participants will be asked to attend their hospital appointment fasting since the previous evening (for purposes of blood and urine biomarker collection).. They will be verbally informed about the assessments by trained research personnel, regarding the nature and purpose of the study, invited to voice any questions or concerns, and given time to decide whether or not to participate. Written informed consent will then be obtained from all study participants prior to any assessment (one copy being given to the participant) and all data will be treated as confidential and anonymised.

Following arrival at Academic Rheumatology and after giving written informed consent, the research professional will collect a fasting urine sample and a 10 ml sample of blood via ante-cubital venepuncture. These samples will be analysed for markers of collagen degradation (e.g. urinary collagen type II crosslinks (CTX-II)) which is predictive of OA progression and markers of generalised inflammation such as serum levels of c-reactive protein (CRP). Other inflammatory markers which are correlated to severity of OA pain, specifically the pro-inflammatory cytokines (e.g. IL-1, IL-6, TNF alpha and the resolvin precursor 12-HDHA) will also be analysed [39]. The research professional will then administer a short structured interview concerning the participant’s recent medical history and current KP status, in case these have altered since completing the baseline questionnaire. They will also administer the General Practice Physical Activity Questionnaire (GPPAQ) which takes less than a minute to complete and allows categorisation of participants into four categories of physical activity: active, moderately active, moderately inactive and inactive.

A number of clinical assessments will then be undertaken in the following sequence:

i) *Hand grip strength*: Each participant will be assessed using a JAMAR hydraulic hand dynamometer (Lafayette Instruments). Participants will be positioned sitting upright in a stable four-legged chair (no armrests) with thighs horizontal and at 90^o^. The assessed arm will be bent with the upper arm vertical, lower arm horizontal, and elbow tight into the waist and the non-assessed arm placed relaxed in their lap. The grip device will then be placed into the participant’s hand and they will be asked to squeeze the device momentarily as hard as possible and then release their grip. This will be performed three times on their dominant hand and the mean of the three readings will be recorded.
ii) *Maximum voluntary quadriceps muscle strength*: The maximum voluntary quadriceps contraction will be assessed for each participant in a standard fashion using the ‘Nicholas Manual Muscle Tester’ (Lafayette Instruments). The participant will be positioned sitting upright on a stable flat surface (no armrests) with thighs horizontal and knees at 90^o^ with their feet raised off the floor. The Muscle Tester will then be positioned at the bottom of the participant’s tibia just above the ankle and they will be asked to push against it as hard as possible in an attempt to raise their lower leg forwards, with resistance provided by the research professional. This will be repeated three times on each leg and the mean value for each side recorded.
iii) *Maximum voluntary hip abductor muscle strength*: The maximum voluntary hip abductor muscle contraction will be assessed for each participant in a standard fashion using the ‘Nicholas Manual Muscle Tester’ (Lafayette Instruments). The participant will be positioned on a clinic couch lying on their side with the lower leg bent for stability and the upper leg held out straight. The Muscle Tester will then be positioned above the ankle of the upper leg and the participant asked to push against it as hard as possible in an attempt to raise their leg up towards the ceiling with resistance provided by the research professional. This will be repeated three times on each leg and the mean value for each side recorded.
iv) *Balance:* Static balance and postural sway either in the medial-lateral or antero-posterior direction will be assessed using the RS Scan force plate. The participant will be asked to stand on the plate looking straight forward for 30 s in two conditions: first with their eyes open and then with eyes closed. Medial-lateral, antero-posterior and total sway will be recorded. Mechanical adaptions in loading are a facet of early knee OA development and progression. Although people with established knee OA demonstrate reduced balance and increased postural sway, these changes may not be evident in the early stages [40] Establishing baseline balance parameters and assessing how these may alter over the course of the cohort study timeline are relevant to understanding the natural history of KP and for early interventions that may delay or prevent worsening symptoms.
v) *Ultrasound (US):* Both knee joints will be imaged using a Toshiba Aplio SSA-770A machine with a multi-frequency (7–12 MHz) linear array transducer. The same equipment and software will be used during the whole study. The assessment will be performed with knee flexion of approximately 20–30° and will include the supra-patellar recess, medial and lateral tibio-femoral spaces. US detected changes will be defined according to definitions accepted by the OMERACT-7 Group [41]. The maximal synovial thickness and effusion depth will be measured in millimetres using the longitudinal axis. These absolute values will be dichotomised as absent (<4 mm) or present (≥4 mm) according to the EULAR Research Group recommendation [42]. A Power Doppler assessment will focus on areas of synovial hypertrophy. A Positive Power Doppler signal which provides information on vascularity will record this feature as absent or present. Only one value per joint will be recorded for each US feature (maximum value across three scanned areas for all participants). It has been previously reported that overall agreement between synovial hypertrophy detected in these three areas of the knee and synovitis detected using the arthroscopy (“gold standard”) was 97% with non-significant difference in sensitivity between three compartments [43].
vi) *Gait Analysis*: Dynamic gait analysis will be performed using the GAITrite portable platform which measures temporal and spatial gait parameters. Participants will be instructed to complete six walks at a natural comfortable pace in their own footwear in a gait laboratory starting a metre before and after the mat to allow a constant speed to be recorded. The six walks will be recorded and will be averaged for final analysis [44]. This includes information on walking speed, cadence, step length, step width and dynamic balance measures (centre of pressure).
vii) *Quantitative Sensory Testing (QST):* Participants will be invited to participate in non-invasive QST which assesses sensitivity to standardised stimuli. This comprises assessment of mechanical pressure pain thresholds (PPT) at baseline and year 1 using a hand-held pressure algometer (Somedic AB, Sweden) that is connected to a computer (HP ProBook 4520 s). The algometer will consist of a rod with a circular end (1cm^2^) that is placed perpendicular to the skin and pressure applied at a gradually increasing rate (standardised rate set at 30 kPa/s) until the participant indicates that the sensation has changed from pressure to pain by pressing a button. The algometer is then immediately taken off the skin. Participants will be familiarised with the test by the research professional who uses the algometer to apply gradual pressure to a fingernail of the dominant hand. As soon as this pressure elicits pain, participants will be asked to press a button to stop the test. This familiarisation procedure is standardised and conducted twice for all participants prior to the PPT test commencing.

One cycle of PPT testing will involve the algometer being used on the following 7 anatomical sites: the sternum (3 cm caudal to the sternal notch); the medial tibiofemoral joint line located medial to the patellar ligament of both knees; the lateral tibiofemoral joint line located lateral to the patellar ligament of both knees; and the proximal shins (both legs) [45]. These sites were chosen to avoid influence of pain from other tissues, for example, muscle, ligaments and tendons. We agree that “muscle-deep pain” is widely addressed using QST approaches in studies of this nature, however, there is little experimental representation of the “deep pain sensation” – a core characteristic of knee pain associated with OA. PPTs on such bony surfaces have been shown to be reproducible and recommended for experimental tests of evoked bone-associated pain. Thus, our approach will provide evidence specific to the “deep pain sensation” across localized, distal and remote sites in our study participants. The PPT cycle will be repeated three times with a 2 min rest period in between each cycle. The PPT will be repeated at follow-up (year 3).

In addition to PPT, temporal summation (TS) also known as wind-up ratio and mechanical sensitivity will be assessed at follow-up (year 1) using a 256 millinewton (mN) weighted pinprick stimulator [24, 45]. The stimulator will be applied perpendicular to the skin, 2 cm distal to the infero-medial border of the patella of the knee to detect a sensation of sharpness or pain. The participant will be asked to rate their pain on an NRS of 0–100 where 0 indicates no pain or sharpness and 100 indicates the most intense pain or sharpness. This rating will be recorded. The stimulator will then be applied to the same site 10 times repeatedly at a rate of 1 per second. At the end of 10 pinpricks, participants will be asked to rate the pain or sharpness using the NRS and this is then recorded. The entire procedure will be repeated twice. The TS will be calculated as the mean pain rating of both series of repetitive pinprick stimuli divided by the mean pain rating of both baseline NRS measures. The mechanical sensitivity will be calculated as the mean pain rating of both baseline NRS measures.

Participants will be familiarised with the tests first on their non- or least affected knee. The tests will then be conducted using their worst or most affected knee. The TS test and mechanical sensitivity test will be repeated at Year 3.

viii) *Radiographs:* Bilateral tibio-femoral and patello-femoral radiographs will be taken using a standardised protocol (standing posterior-anterior (PA) and skyline views) and scored by two specifically trained raters (GSF and AS). A Perspex Rosenberg template with lead beads will be used for the standing PA view to standardise the degree of knee flexion, foot rotation and magnification [46]. PA radiographs will be taken with the patient facing the x-ray tube while standing on the Rosenberg template and leaning forwards with their thighs touching the anterior aspect of the apparatus, the x-ray beams passing from the posterior aspect through to the anterior aspect of the knee. Variable jigs will be used for the skyline view to obtain 30^0^ of knee flexion with the participant lying in a reclined supine position on a couch. Grading of radiographs for changes of OA will include [1] the summated Kellgren and Lawrence (KL) score and [2] the Nottingham logically devised line drawing atlas (NLDA) for individual scoring of osteophyte (0–5) and joint space width (−1 to +5, using sex-specific atlases) for each medial tibio-femoral (TF), lateral TF and patello-femoral (PF) compartment similar to previous published epidemiological studies [23].

**Fig. 1:**
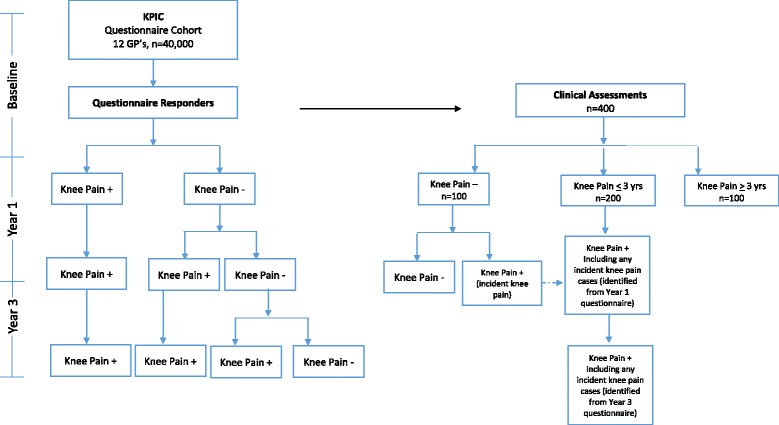
Recruitment Flowchart for KPIC. Questionnaire: administered at baseline, Year 1 and Year 3 for all who indicated interest and consent to follow up at baseline. Clinical Assessments: will be repeated for early Knee Pain (and new knee pain cases from the no knee pain groups). No repeat assessments will be undertaken for the establish Knee Pain group

The flow of participants through the study is depicted in Fig. 1.

#### Year 1 follow-up

### Questionnaire

Of those participants who indicated interest and consent to follow-up at baseline, a follow-up postal questionnaire will be issued in year 1. The year 1 questionnaire will follow the format of the baseline questionnaire with some changes. We will not re-administer questions on constitutional risk factors such as early knee alignment and 2D4D ratio since these would not change from baseline. We will add a set of questions on sleep from the Medical Outcome Survey. A modified body pain mannequin (see Additional file 2) with two questions on symptom severity and widespread pain index will also be included to allow scoring of fibromyalgia as a diagnosis with binary (present/absent) classification using a validated tool [47]. The questionnaire will also be updated to include the Life Orientation Test (LOT) which evaluates a lack of positive beliefs as well as low negative beliefs [48] and a short item test on conscientiousness (Big 5) [49]. We will also add an illness perceptions questionnaire to measure patient’s belief about their KP [50]. These validated tools will be included as they contribute to the psychosocial aspects of the disease and could be predictors of patient and clinical outcomes. All questionnaire changes will be approved following feedback and review from PPI groups and subsequently, submitted for NREC approval.

### Clinical assessment

From the responders to the Year 1 questionnaire, any new cases of early KP (incident KP for most days in a month for the past 12 months) will be identified and invited for clinical assessments as per the baseline protocol. It is estimated that the new incident KP cases may number from *n* = 70–110 from the Year 1 questionnaire mail-outs (based on an annual incidence of 3.2%) [23].

For those early KP participants who attended the baseline appointment, there will be repeat assessments of muscle strength, gait, balance and ultrasound. The PPT assessments will be done on the most painful knee of participants with KP. For those who have an equal pain severity in both knees, the right knee will be assessed. The PPT protocol will also be complimented by TS testing. TS or windup will be used to assess central sensitivity and characterise central pain processing abnormalities by measuring how pain is perceived (using a NRS) once repetitive stimuli (weighted pinprick) is applied [51, 52].

#### Year 3 follow-up

A follow-up questionnaire will also be mailed out at Year 3. Only the early KP group (including incident KP identified at year 1) will be re-assessed at year 3 for change of outcomes. In addition, people without KP at baseline or year 1 but reporting KP at year 3 will be considered as new incident KP cases and will be assessed clinically (including knee radiographs) as per the baseline protocol.

### Sample size

The sample size calculated was based on the primary objective of this study: to examine the natural history of KP within a community setting which involves the recruitment of as many knee pain positive and knee pain negative individuals with detailed exposure data captured from the questionnaire. The subsequent objectives are to examine the incidence, progression and associated risk factors.

1. Source population: 40,000 questionnaires will be sent via post with a target of recruiting 10,000 participants (response rate 25%), which will form the source population of this study. Based on our previous studies, this would give 2500 people with knee pain (KP +) and 7500 people without knee pain (KP -). The sample size is 10 times larger than the subsequent sample size calculations for incidence, progression and risk factors of KP.
2. Sample size for incidence and progression of KP: A sample of 730 participants will allow the detection of 3% (±1%) annual incidence of knee pain in a population over the age of 45^24^ at power of 90% (alpha 0.05, two-sided). This sample will also be able to detect a 14% occurrence of knee pain progression (worsening) (±5%) 24 at a power of 90% (alpha 0.05). An annual drop off rate of 30% plus 3% annual incidence rate of KP is excluded from the source population.
3. Risk factors: A logistic regression model was used to calculate the sample size for one primary risk factor and multiple covariates. According to the annual incidence of 3% KP^24^ and an OR of 2 associated with overweight/obese [53] and assuming a multiple correlation coefficient of other covariates is 0.3, 702 participants are required for this risk factor analysis to give a power of 90% (alpha 0.05). For progression (i.e., 14% KP worsening), however, 203 participants are required.

### Sub-studies sample sizes

Sample size for NP: According to a NP prevalence of 28% (±8) based on a previous community population sample [15], 85 participants are required to yield a power of 90% (alpha 0.05).

Sample size for PPT: According to a difference of 0.32 SD (standard deviation) on pressure pain threshold (PPT) between established KP (group ii) and No KP [54] and 0.16 SD between early KP and no KP, 600 participants are required for a three-group comparison with an unbalanced (400:100:100 for the “Early KP”, “Established KP” and “No KP”) one-way ANOVA design. This unbalanced design will be applied to keep the main interest on early KP and its subsequent follow-up for progression.

Sample size for US: Data from a previous Nottingham community based case control study was used and this study [55] comprised four groups: people with KP and radiographic changes (Kellgren and Lawrence (K&L) score ≥ 2); people with KP without radiographic changes; people with no KP but radiographic changes; and people with no KP and normal x-rays. We used KP only as “early KP”, KP plus radiograph changes as “established KP” and combined the two no KP groups to form the control. We then calculated standardised effect sizes for each case group versus control based on means/SD (1.06, 2.02 and 0.96, respectively). The total sample size required is 80 (group balance 40:20:20) which will yield 90% power with 5% type I error for this unbalanced multiple group case-control study.

### Statistical analysis

#### Questionnaire and clinical assessments

For baseline questionnaire data, prevalence of KP and type of KP (e.g., NP) will be estimated. Risk factors associated with KP and different types of KP (e.g., NP) will be examined. Odds ratio (OR) and 95% confidence interval (CI) will be given to present the association and logistic regression model will be used to adjust for confounding factors such as age, gender, body mass index and pain severity.

For the baseline clinical assessment data, the overall difference among three groups categorised according to KP status (Early KP, established KP and controls) will be analysed using multinomial logistic regression. This is an extension of binary logit regression when the categorical dependent variables have more than two response categories. The “No KP” group will be chosen as a reference (base). As cases and controls will be frequency matched (by age and gender) unconditional logistic regression analysis will be chosen. The odds ratio (OR) and 95% CI will be used to measure an association. All models will be adjusted for potential confounding factors and checked for interactions and collinearity as appropriate.

The loss to follow-up rate (from baseline to Year 1 and then to Year 3) can be measured in terms of variables such as age, gender and severity of symptoms and in order to mitigate the differential bias, a multiple imputation model alongside sensitivity analyses will be considered. For follow-up data, incidence and progression of KP will be estimated. Logistic regression model will be used for year 1 and other single time point follow-up analysis. The Kaplan–Meier method will be used to generate survival curves and a log-rank test will be used to examine the time-to-event outcomes for multiple follow-up data. The proportional hazards assumption will be examined graphically using the Kaplan–Meier method for each risk factor. The Cox proportional hazards model will be used to calculate the hazard ratio (HR), adjusted for confounding factors such as age, gender and BMI. Differences between the study population; those invited to participate and responders will be tested using a one-way analysis of variance (ANOVA) with post hoc Bonferroni correction. Statistical significance will be inferred when *P* value is less than 0.05, or when the 95% confidence interval (CI) does not include unity.

#### KP phenotypes

We hypothesise that phenotypic markers representative of underlying pain mechanisms can be identified using self-report questionnaire items. Groups of questionnaire items will be entered into an exploratory structural equation model (ESEM) in order to identify underlying (latent) constructs (e.g. depression) being measured. ESEM also specifies the strength of association between each item and the underlying construct. Items showing the strongest association (*p* < 0.05) to each underlying construct will be shortlisted for inclusion within a developing tool which aims to classify underlying pain mechanisms in individuals reporting knee OA pain. ESEM will be conducted using M*Plus* 7.

#### Reproducibility

For reproducibility of assessments, we will use the kappa-statistic for categorical data (x-rays and dichotomous US data) and the concordance correlation coefficient for continuous data. The magnitude of agreement on categorical data will be measured using the unweighted kappa statistic (for binary) or the weighted kappa statistics (for ordered categorical data) [56]. A numerical rating of kappa will be interpreted according to accepted criteria (0–0.2: slight; 0.2–0.4: fair; 0.4–0.6: moderate; 0.61–0.8: substantial; 0.81–1.0: almost perfect) and 95% confidence intervals will be reported [57]**.**

All analyses will be undertaken using Stata Statistical Software: Release 13 (StataCorp. 2013, College Station, TX: StataCorp LP). *P* values less than 0.05 will be considered significant.

## Discussion

A population-based prospective cohort study is needed to determine the natural history of KP, involving affected and unaffected people at baseline, to examine the incidence, progression, different KP phenotypes and associated risk factors and outcomes. Results from our study can provide an insight into possible pain phenotypes according to self-reported factors such as intermittent versus persistent KP, localised versus generalised KP, qualitative descriptors of KP that suggest central sensitisation, and presence of multiple regional pain.

The advantages of KPIC in comparison with other studies published so far include the focus on the entire spectrum of knee pain, the long follow-up duration (three years in the first instance), the repeated measures of both exposures and outcomes at several time points and the battery of clinical assessments in people with early KP, no KP and established KP. It is important that the KPIC study population is representative of the general population (See Additional file 3). The inclusion criteria is set slightly younger (40 years and above) than other population cohorts such as the Genetics of OA and Lifestyle study (45 years and over) and the British National Survey (55 years and over) in order to capture more participants with any symptoms or radiographic signs of early OA as opposed to established KP which has already been extensively researched but mainly in people over 50 [58–62].

The expected response rate of 25% is lower than the mean response rate of 60% for postal surveys published in medical journals [63]. However, this is based on previous surveys to the community in Nottingham and this rate could be improved with the use of reminder letters and an accompanying postal questionnaire. The response rate could also be improved with the use of financial incentives, however, there is limited evidence to support this [64]. In order to retain participants through the course of the KPIC timeline, the authors will ensure timeline questionnaire reminders (4–6 weeks) post initial questionnaire mail out as well as annual newsletters with updates on study progression such as successful recruitment to a sub-study and preliminary findings.

There are some limitations to the study design of KPIC. Firstly, being a questionnaire-based cohort study, the design is prone to a number of biases including response bias (participants with KP more likely to respond to the questionnaire) and recall bias (participants with KP may recall events and exposures more accurately compared to those without KP). However, the question on KP is very specific and focused on any symptoms in and around the knee for most days of at least one month in the previous 12 months which is the ‘gold standard’ for epidemiological research studies [30]. Current knee pain was defined as pain in or around the knees for most days of the past one month for incident knee pain. This was different from early onset knee pain which was defined as knee pain (ever or current for most days of at least a month) in the past 3 years. However, due to the transient nature of knee pain, it is possible that the baseline questionnaire might not allow us to correctly identify participants as being knee pain positive or knee pain negative (misclassification bias). A self-reported questionnaire approach also ensures the absence of interview bias from the study results [23]. Secondly, although the questionnaire cohort is large (*n* = 40,000), the clinical subset cohort is relatively small (*n* = 400). There may be limitations to using multiple adjusted effect estimates that have all been taken from a single logistic regression model. A bias otherwise known as the table 2 fallacy could occur where the interpretation of the confounder estimates may be different than for the exposure effect estimates [65]. Thirdly, there is a selection bias associated to a potential differential loss to follow-up in the group with moderate-severe pain or participants who are frail. The responders and non-responders will be compared at Year 1 and Year 3 in order to measure and subsequently adjust for this bias.

Knowledge obtained from this proposed cohort study could be important for understanding the natural history of KP, including incidence, in a community-based population setting. Currently, we are unaware of any other population-based cohort established for people at risk for KP, therefore this is the first cohort to cover the full natural history from no KP to KP, then to the outcomes of KP. In addition, we are not only interested in KP severity but also KP phenotypes. Identifying specific subgroups or phenotypes of people with KP and possible novel associations with each phenotype are important as it may help to develop specific individualised treatment or management strategies. The cohort will also help us to understand the implications of central sensitisation versus localised pain features and use this to improve the diagnosis, treatment and management of KP and knee OA in the general population.

## Additional files

Additional file 1: High Risk Occupation Categories (DOCX 17 kb)

Additional file 2: Modified body pain mannequin used to identify fibromyalgia (DOCX 163 kb)

Additional file 3: Comparison of demographics between KPIC and other UK based studies (DOCX 12 kb)

## Abbreviations

2D4D: 2ND digit and 4th digit ratio
ANOVA: Analysis of Variance
AS: Aliya Sarmanova
AV: Ana Valdes
CCC: Concordance Correlation Coefficient
CFI: Comparative Fit Index
CI: Confidence Interval
CM: Centimetre
CPM: Central Pain Modulation
CRP: C reactive protein
CTX-II: C-telopeptide of type II collagen
DNIC: Descending noxious inhibitory control
DW: David Walsh
EPIC: European Prospective Investigation into Cancer study
ESEM: Exploratory Structural Equation Model
GOAL: Genetics of Osteoarthritis and Lifestyle study
GP: General Physician
GPPAQ: General Practice Physical Activity Questionnaire
GSF: Gwen Sascha Fernandes
HADS: Hospital Anxiety and Depression Scale
HDHA: Hydroxy-docosahexaenoic acid
HFCS: High Fructose Corn Syrup Study
HSE: Health Survey of England study
ICOAP: Intermittent and Constant Osteoarthritis Pain
IL: Interleukin
KA: Kehinde Akin
KL: Kellgren Lawrence
KP: Knee Pain
KPIC: Knee Pain in the Community
LM: Laura Marshall
LOT: Life Orientation Test
MD: Michael Doherty
mN: Millinewton
NF: Nadia Frowd
NLDA: Nottingham Line Drawing Atlas
NREC: Nottingham Research Ethics Committee
NRS: Numerical rating scale
OA: Osteoarthritis
OR: Odds Ratio
PA: Posterior-anterior
PCS: Pain Catastrophising Scale
PDQ: Pain Detect Questionnaire
PF: Patellofemoral
PPT: Pain Pressure Thresholds
QST: Quantitative Sensory Testing
ReR: Response Rate
TF: Tibiofemoral
TNF: Tumor Necrosis Factor
TS: Temporal summation
Tucker Lewis Index: Tucker Lewis Index
WZ: Weiya Zhang
