# Supplementary material for: Knee pain and related health in the community study (KPIC): a cohort study protocol
Source: BMC Musculoskelet Disord. 2017 Sep 21;18:404. doi: 10.1186/s12891-017-1761-4 (PMC5609004; doi:10.1186/s12891-017-1761-4)
Supplement: Supplementary file 1 — High Risk Occupation Categories (DOCX 17 kb) [file 12891_2017_1761_MOESM1_ESM.docx]

| **Agreed High Risk Category** | **Filter Searches** |
| --- | --- |
| Auto-Industry/Mechanic | Car Fitter, Car Seat Assembly, Paint Sprayer/Car Repair (seat assembler, reconditioning, painting), car mechanic, car repairer, Panel beater |
| Tradesmen: Construction | Brick, Build, Property, Construction, Scaffold, Damp, Ground worker, Quarry worker/operative, roof, Joiner, Carpenter, Wood, Fitter, Sign maker, Furniture design and manufacture, Upholsterer, Cabinet Maker, Shop Fitter, Window, Glazing, Glazier, Double Glazier |
| Tradesmen: Other | Electric, Jointer Electrical, Lineman, Panel wirer/wireman, Carpet, Floor, Slab, Paint, Plaster, Cabinet Sprayer, Plumber, Pipe Fitter |
| Emergency Services | Police, Firefighter, Army, Soldier, National Service, RAF, Marines, Navy, Naval, Paratrooper, Royal (air force etc.), Ambulance, Paramedic, Military, WRNS ( Women’s Royal Navy Service) |
| Factory/Warehouse Worker | Factory, Workshop, Worker, Assembly, Production, Warehouse, Picker, Packer |
| Land management/Cultivation | Agriculture, Agricultural, Farm, Farmer, “Picker (fruits)”, Herdsman, Dairy man, Gardener, Green, Landscape, Lawn, Horticulture, Groundsman, Greenkeeper, Horticulturalist, Timber yard worker, forest worker, forester, forestry |
| Manual Workers Engineering | Engineer, Millwright, Lagger (Insulators)/ Lagging |
| Health Services | Nurse, Nursing, carer, care worker, care assistant, support worker, Home help, RGN (registered general nurse), RNMH (registered nurse mental health), Ward Orderly, SRN (State Registered Nurse), RMN (Registered Mental Nurse), RNLD |
| Coal Miner | Coal, Mine, Colliery, Down pit, Mining, Collier |
| Metal Worker | Steel, Weld, Metal , Blacksmith, Foundry, Moulder, Plater, Fabricator |
| PCM (Porters, Cleaners, Maintenance) | Porter, Clean Window, Cleaner, Valeter, Wash (Car), Janitor, Domestic Assistant, Chambermaid, Hospital domestic, domestic, caretaker, building maintenance, maintenance, handyman, building assistant |
| PE Teacher | PE, Physical |
| Postman | Post, mail |
| Labourer | Labourer, Pipe yard Worker, Concrete, Installer, Mason, Fibre glass, Asphalt(er), Digging trenches, Fencer, Manual |
| Road Worker | Roadworks, Road, Highway, Platelayer, Tarmac layer, Tarmacer |
| Shipyard Worker | Shipwright |
| Sports-Other | Any , Climbing instructor, Parachute instructor |

References:

*Coggon,* D., Croft, P., Kellingray, S., Barrett, D., McLaren, M. and Cooper, C. (2000), Occupational physical activities and osteoarthritis of the knee. Arthritis & Rheumatism, 43: 1443–1449. doi:10.1002/1529-0131(200007)43:7<1443::AID-ANR5>3.0.CO;2-1

*Andersen S,* Thygesen LC, Davidsen M*, et al* Cumulative years in occupation and the risk of hip or knee osteoarthritis in men and women: a register-based follow-up study, Occup Environ Med Published Online First: 11 January 2012. doi: 10.1136/oemed-2011-100033

*Industrial Injuries Advisory Council,* Disorders of the Knee. 17^th^ of May 1995.

*Palmer KT.* Occupational activities and osteoarthritis of the knee. Br Med Bull. 2012;102:147-70.
