# Supplementary material for: Knee pain and related health in the community study (KPIC): a cohort study protocol
Source: BMC Musculoskelet Disord. 2017 Sep 21;18:404. doi: 10.1186/s12891-017-1761-4 (PMC5609004; doi:10.1186/s12891-017-1761-4)
Supplement: Supplementary file 2 — Modified body pain mannequin used to identify fibromyalgia (DOCX 163 kb) [file 12891_2017_1761_MOESM2_ESM.docx]

We are still interested in your body pain experience particularly in the past one week. Please can you think of your pain and symptoms but exclude any that have been due to known illnesses such as arthritis, lupus etc.

a) In the diagrams below, please tick the boxes where you have experienced pain in the region indicated by the shaded areas. Please tick all relevant boxes (the boxes are either placed in the area itself or joined to the area by a line).


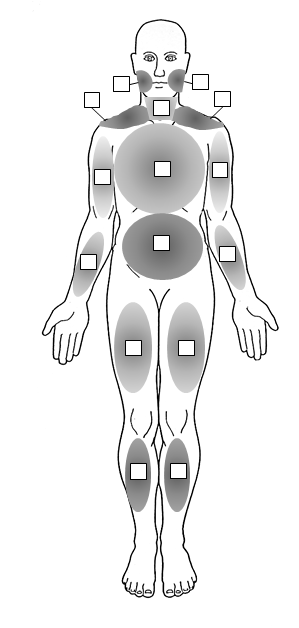

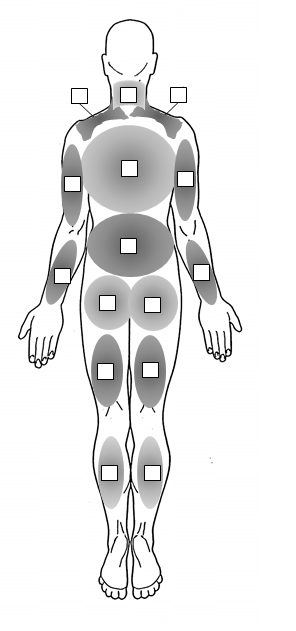


b) Please could you indicate in the tick boxes your level of symptom severity score over the past week using the following scale.

0 = No problem 1 = Slight or mild 2= Moderate; 3= Severe:

problems; generally considerable pervasive,

mild or intermittent problems; often continuous, life

present and/or disturbing problems

at a moderate level

a) Tiredness

b) Waking

Unrefreshed

c) Concentration

(Forgetfulness

and problem

solving)

c) Lastly, which of the following symptoms have you experienced in the past 1 week? Please tick all that apply.

Muscle pain

Muscle weakness

Numbness/tingling

Fever

Irritable bowel syndrome

Pain/cramps in abdomen

Constipation

Pain in upper abdomen

Nausea

Diarrhoea

Dry mouth

Vomiting

Heartburn

Oral ulcers

Loss/change in taste

Loss of appetite

Fatigue/Tiredness

Thinking/ problems remembering

Headache

Dizziness

Depression

Nervousness

Blurred vision

Fits

Chest pain

Wheezing

Shortness of breath

Ringing in ears

Dry eyes

Hearing difficulties

Itching

Raynaud’s*

Hives**

Rash

Sun sensitivity

Easy bruising

Hair loss

Frequent urination

Painful urination

Bladder pain
