# Supplementary material for: Knee pain and related health in the community study (KPIC): a cohort study protocol
Source: BMC Musculoskelet Disord. 2017 Sep 21;18:404. doi: 10.1186/s12891-017-1761-4 (PMC5609004; doi:10.1186/s12891-017-1761-4)
Supplement: Supplementary file 3 — Comparison of demographics between KPIC and other UK based studies (DOCX 12 kb) [file 12891_2017_1761_MOESM3_ESM.docx]

|  | **KPIC** | **GOAL* Controls** | **HSE***  **1996** | **British National Survey** | **Prostate Cancer Controls** | | **HFCS** | **Norfolk EPIC** |
| --- | --- | --- | --- | --- | --- | --- | --- | --- |
| **Male**  Age (Range) | 62.96  (41-86) | 66.2  (45 – 81) | 65 - 74 | 55 – 64 | 68.3  (44 -77) | 66 | | 65 – 74 |
| Mean BMI | 27.49 | 27.7 | 27 | N/C | N/C | 26.8 | | 26.7 |
| **Female**  Age | 61.54  (41-83) | 61.8  (45-81) | 65 -74 | 55-64 | N/C | 26.7 | | 26.6 |
| Mean BMI | 27.24 | 27.3 | 27.3 | N/C | N/C | 26.7 | | 26.6 |

*GOAL: Genetics of Osteoarthritis and Lifestyle study; HSE: Health Survey of England study; HFCS: High Fructose Corn Syrup Study; EPIC: European Prospective Investigation into Cancer study
